# Yeast importin-β is required for nuclear import of the Mig2 repressor

**DOI:** 10.1186/1471-2121-13-31

**Published:** 2012-11-06

**Authors:** Alejandra Fernández-Cid, Montserrat Vega, Pilar Herrero, Fernando Moreno

**Affiliations:** 1 Department of Biochemistry and Molecular Biology, University of Oviedo, 33006, Oviedo, Spain; 2 Present Address: MRC Clinical Sciences Centre, Imperial College Faculty of Medicine, London, UK

## Abstract

**Background:**

Mig2 has been described as a transcriptional factor that in the absence of Mig1 protein is required for glucose repression of the *SUC2* gene. Recently it has been reported that Mig2 has two different subcellular localizations. In high-glucose conditions it is a nuclear modulator of several Mig1-regulated genes, but in low-glucose most of the Mig2 protein accumulates in mitochondria. Thus, the Mig2 protein enters and leaves the nucleus in a glucose regulated manner. However, the mechanism by which Mig2 enters into the nucleus was unknown until now.

**Results:**

Here, we report that the Mig2 protein is an import substrate of the carrier Kap95 (importin-β). The Mig2 nuclear import mechanism bypasses the requirement for Kap60 (importin-α) as an adaptor protein, since Mig2 directly binds to Kap95 in the presence of Gsp1(GDP). We also show that the Mig2 nuclear import and the binding of Mig2 with Kap95 are not glucose-dependent processes and require a basic NLS motif, located between lysine-32 and arginine-37. Mig2 interaction with Kap95 was assessed *in vitro* using purified proteins, demonstrating that importin-β, together with the GTP-binding protein Gsp1, is able to mediate efficient Mig2-Kap95 interaction in the absence of the importin-α (Kap60). It was also demonstrated, that the directionality of Mig2 transport is regulated by association with the small GTPase Gsp1 in the GDP- or GTP-bound forms, which promote cargo recognition and release, respectively.

**Conclusions:**

The Mig2 protein accumulates in the nucleus through a Kap95 and NLS-dependent nuclear import pathway, which is independent of importin-α in *Saccharomyces cerevisiae*.

## Background

In eukaryotic cells the confinement of the genetic material and the transcriptional machinery to the nucleus allows the regulation of central processes including gene expression, signal transduction and control of the cell cycle. One of the key features of such regulation is the selective bi-directional transport of several proteins between the cytoplasm and the nucleus. Such transport happens through large membrane structures termed nuclear pore complexes (NPCs), comprised of approximately 30 proteins collectively termed nucleoporins (Nups) [1]. The mechanism of translocation through the pore is not well understood, but is facilitated by a number of nuclear transport factors or carrier proteins termed karyopherins [2]. In higher eukaryotes, as well as in the yeast *S*. *cerevisiae*, the karyopherins that mediate nuclear import of proteins and RNAs are generally known as importins [3], whereas those mediating nuclear export are known as exportins [4]. Importins associate with their macromolecular cargo in the cytoplasm, either directly or indirectly via adaptor proteins [5]. Cargo release is achieved by association of the importins with the GTPase Ran (Gsp1 in yeast) in the GTP-bound form (Gsp1-GTP) [6]. Nuclear export is essentially the reciprocal process, with cargo recognition occurring in the nucleus in the presence of Gsp1(GTP) and cargo complexes dissociating in the cytoplasm upon GTP hydrolysis. Gsp1 is present at high concentrations in the nucleus in the GTP-bound form, and mainly in the GDP-bound form in the cytoplasm [7]. This compartmentalization depends on the localization of the proteins that regulate the nucleotide state of Gsp1. GTP hydrolysis requires a GTPase-activating protein (GAP) that is present in the cytoplasm. Conversely, the exchange of GDP to GTP is catalyzed by a guanine nucleotide exchange factor (GEF) that is bound to the chromatin [1,8,9]. Importin-β (Kap95 in yeast) was first identified as the transport factor for proteins carrying classical nuclear localization signals (NLSs), such as those of nucleoplasmin or the SV40 T antigen [10]. Importin-β hardly ever binds these classical NLSs directly, but binds importin-α (Kap60 in yeast), which in turn binds these NLSs [5]. While this pathway is termed the classical nuclear import pathway, several alternative import pathways have been characterized. Most karyopherins bind their cargos directly, and even importin-β, in some cases, is able to recognize cargo substrates without the need for an importin-α (Kap60) adaptor protein, as in the case of ribosomal proteins [11], SREBP- 2 [12], cyclin B1 [13], SRY [14], PTHrP [15] or yeast phosphatidylinositol 4-kinase [16].

Mig2 was identified several years ago as a repressor that collaborates with Mig1 to cause glucose-induced repression of the *SUC2* gene [17]. A genome wide expression profiling survey of the yeast genome revealed that all the genes regulated by Mig1 are also regulated by Mig2. Thus, it was postulated that Mig2 always works in conjunction with Mig1 [18]. However, Mig1 appears to be the essential component, since its presence is necessary and sufficient to cause full repression of most genes even in the absence of Mig2. Mig1 is a zinc-finger protein that binds to the promoters of many genes repressing their transcription [19]. Mig1 promoter binding is regulated by Hxk2 and Snf1 proteins. Hxk2 has a regulatory role in glucose repression and this is dependent on its interaction with Mig1. Together, they form a repressor complex located in the nucleus of *S*. *cerevisiae*. In high glucose, nuclear Hxk2 stabilizes the complex by blocking Mig1 S311 phosphorylation by Snf1 protein kinase and this is important for glucose repression signaling [20-22]. In low-glucose, Hxk2 serine-14 and Mig1 serine-311 phosphorylation trigger their export from the nucleus by the Xpo1(Crm1) and Msn5-dependent pathways, respectively [21,23,24]. Thus, Hxk2 and Mig1 become mainly cytoplasmic [20] and this initiates the expression of several genes regulated by glucose repression.

Recently, it has been described that Mig2 moves between the nucleus and mitochondria in a glucose-dependent manner [25]. However, the mechanisms by which Mig2 enters into and leaves the nucleus are largely unknown. Since Mig2 is too large to translocate through the NPC by diffusion, its transport across the nuclear envelope is probably mediated by carrier proteins. In this study, we show that the Mig2 protein is an import cargo of the importin-β (Kap95) carrier in *S*. *cerevisiae*. We also show that Mig2 interacts with Kap95 in the presence of Gsp1(GDP) without the participation of other auxiliary proteins. Moreover, Mig2-Kap95 interaction depends on a Mig2 NLS motif located between lysine-32 and arginine-37. The observed Mig2-Kap60 interaction is not required for Mig2 entry into the nucleus and does not require the NLS motif of the Mig2 protein. We also demonstrate that Mig2 is recognized by Kap95 in the cytoplasm in the presence of Gsp1-GDP and cargo complexes dissociate in the nucleus upon the GDP/GTP exchange in the Gsp1 protein.

## Methods

### Strains and growth conditions

The *S*. *cerevisiae* strains used throughout this study were derived from W303-1A [26] and BY4742 [27] haploid wild-type strains and are listed in Table 1. Strains FMY501, FMY527, FMY528 and FMY535 expressing Mig2-GFP were constructed respectively by homologous recombination in W303-1A *kap95*^*ts*^, *kap60*^*ts*^ and *Δmsn5* strains using a GFP-HIS3 tagging cassette obtained from pFA6a-GFP-HIS3 plasmid [28]. We created two NLS’s mutant alleles of *MIG2* by mutating the *MIG2* gene in the pGEMT-MIG2-GFP/HIS3 plasmid to generate *MIG2K32AK33AR37A* (encodes Mig2*nls1* protein) and *MIG2R75AR76AK79A* (encodes Mig2*nls2* protein) genes using a PCR-based mutagenesis protocol. Then, by using these constructs, recombination cassettes were obtained by PCR; these cassettes contained the mutated *MIG2* locus and the *HIS3* marker. These linear DNAs were integrated into the *MIG2* locus of strain FMY501. To confirm the correct insertion, we PCR amplified the *MIG2* locus from strains: FMY523, contains the *MIG2* gene replaced by the *MIG2nls1* gene and FMY524, contains the *MIG2* gene replaced by the *MIG2nls2* gene, to detect the presence of the mutant *MIG2K32AK33AR37A* and *MIG2R75AR76A*, *K79A* alleles by sequence analysis respectively.

**Table 1:** ***Saccharomyces cerevisiae *strains used in this study**

**Name**	**Relevant genotype**	**Source/Ref.**
W303-1A	*Mat α ura3-52 trp1-289 leu2-3,112 his3-Δ1 ade2-1 can1-100*	[26]
BY4742	*MATα his3Δ 1 leu2Δ 0 met15Δ 0 ura3Δ 0*	[27]
DBY2052	*Mat α hxk1:: LEU2 hxk2-202 ura3-52 leu2-3,2-112 lys2-801 gal2*	[29]
THG1	MATα *leu2-1 ura3-1 lys1-1 hxk1:: LEU2 hxk2:: LEU2 glk1:: LEU2*	[30]
H174	*MATα ade2-1 canJ-100 his3-11,15 leu2-3,112 trp1-1 ura3-1 mig1-6 J:: LEU2*	[31]
MAP24	*MATa mig1::loxp mig2::loxp-KAN-lox can1-100 his3-11,15 leu2-3,112 trp1-1; ura 3-1*	[32]
JCY1407	*MATa ade2-1 ura3-1; his3-11,15 trp1-1 leu2-3,112 can1-100 kap95:: HIS3 pSW509/pkap95-L63A*	[33]
JCY1410	*MATa ade2-1 ura3-1 his3-11,15 trp1-1 leu2-3,112 can1-100 srp1-31*	[33]
TetR-GFP	*MATa ade2-1 his3-11,15 leu2-3,112 trp1-1 ura3-1 tetOx200:: URA3 tetR-GFP:: LEU2*	[34]
Y03694	*MATα his3Δ1 leu2Δ0 met15Δ0 ura3Δ0 msn5::kanMX4*	Euroscarf
Y14575	*MATα his3Δ1 leu2Δ0 lys2Δ0 ura3Δ0 mig2::kanMX4*	Euroscarf
FMY501	*MATa ade2-1 his3-11,15 leu2-3,112 trp1-1 ura3-1 MIG2:: GFP*	This work
FMY523	*MATa ade2-1 his3-11,15 leu2-3,112 trp1-1 ura3-1 MIG2*_*NLS1*_*:: GFP*	This work
FMY524	*MATa ade2-1 his3-11,15 leu2-3,112 trp1-1 ura3-1 MIG2*_*NLS2*_*:: GFP.*	This work
FMY525	*MATα; ade2-1; canJ-100; his3-11,15; leu2-3,112; trpl-J; ura3-1; mig1-6 J:: LEU2; MIG2*_*NLS1*_*:: GFP.*	This work
FMY526	*MATα ade2-1 canJ-100 his3-11,15 leu2-3,112 trp1-1; ura3-1; mig1-6 J:: LEU2; MIG2*_*NLS2*_*:: GFP.*	This work
FMY527	*MATa ade2-1 ura3-1 his3-11,15 trp1-1 leu2-3,112 can1-100 kap95:: HIS3 pSW509/pkap95-L63A MIG2:: GFP.*	This work
FMY528	*MATa ade2-1 ura3-1 his3-11,15 trp1-1 leu2-3,112 can1-100 srp1-31 MIG2:: GFP.*	This work
FMY535	*MATα his3Δ1 leu2Δ0 met15Δ0 ura3Δ0 msn5::kanMX4 MIG2:: GFP*	This work
FMY536	*MATa ade2-1 his3-11,15 leu2-3,112 trp1-1 ura3-1 tetOx200:: URA3 tetR-GFP:: LEU2 mig2::kanMX4*	This work

*Escherichia coli* DH5*α* (*F Ø80dlacZ ΔM15 recA1 endA1 gyrA96 thi*-*1 hsdR17*(*rk*-*rk*-) *supE44 relA1 deoRΔ 99U169*) was the host bacterial strain for the recombinant plasmid constructions.

Yeast cells were grown in the following media: YEPD, high-glucose (2% glucose, 2% peptone, and 1% yeast extract), YEPGly, low-glucose (0.05% glucose, 3% glycerol, 2% peptone, and 1% yeast extract) and synthetic media containing the appropriate carbon source and lacking appropriate supplements to maintain selection for plasmids (2% glucose -SD, high glucose- or 3% glycerol and 0.05% glucose -SGly, low glucose-) and 0.67% yeast nitrogen base with ammonium sulfate and without amino acids. Amino acids and other growth requirements were added at a final concentration of 20-150 μg/ml. The solid media contained 2% agar in addition to the components described above.

### Plasmids

To construct the yeast expression plasmid pGEMT/MIG2-GFP-HIS3 the yeast strain FMY501 was used as template in conjunction with oligonucleotides MIG2-d: TAAGCTGTGGCGATGTGCTG and MIG2-r: CCACCTTATCTCCACGGGAA in a PCR based protocol. To create the *MIG2* mutant alleles *MIG2nls1K32AK33AR37A* and *MIG2nls2R75AR76AK79A* the plasmid pGEMT/MIG2-GFP-HIS3 was used as template in conjunction with oligonucleotides MIG2nls1-d: GTGGTTTCCATCGGTTAGAACA TGCAGCGAGAACACTTGGCAAGACACACTGGGGAAAAACCTC, MIG2nls1-r: GA GGTTTTTCCCCAGTGTGTTGCCAAGTGTCTCGCTGCATGTTCTAACCGATGGAA ACCAC, MIG2nls2-d: AACGCATACAGGGCAACTCAAGCGGCATTGAAGGCAGC TAGCGTACAGAAACAGGAGTT and MIG2nls2-r: AACTCCTGTTTCTGTACGCTA GCTGCCTTCAATGCCGCTTGAGATTGCCCTGTATGCGTT, in the PCR based site-directed mutagenesis method [35] to generate plasmids pGEMT/MIG2nls1-GFP-HIS3 and pGEMT/MIG2nls2-GFP-HIS3. All nucleotide changes were verified by DNA sequencing. The yeast expression plasmid YEp352/Hxk2*nes2*(Ala)-GFP was constructed as indicated previously [36].

GST fusion vectors pGEX/MIG2 and pGEX/MIG2nls1 were constructed by cloning in the *Sal*I site of pGEX-4 T-1 (GE Healthcare) a previously *Sal*I digested PCR fragment obtained by using pGEMT/MIG2-GFP-HIS3 and pGEMT/MIG2nls1-GFP-HIS3 respectively as templates in conjunction with oligonucleotides MIG2-BD/AD-d: TTCCCGGGTCGACTCATGCCTAAAAAGCAAACGAATTTCCCAGTA and MIG2-BD/AD-r: TTCCCGGGTCGACTCAACTCTTTTGGGACCGTTCAAAACAT. Plasmid pGEX/GSP1 for expression of GST-Gsp1 in *E*. *coli* was a gift from E. Hurt’s laboratory [37] and plasmid pGEX-Kap95 and pGEX-Kap60 for expression of GST-Kap95 and GST-Kap60 in *E*. *coli* was a gift from M. P. Rout's laboratory [38].

pRS316-Su9-RFP a *CEN*-*URA3* plasmid expressing RFP fused to the presequence of subunit 9 of the F_o_-ATPase of *Neurospora crassa* under control of the *ADH1* promoter was a gift from P. Sanz.

### Fluorescence microscopy

Yeast strains expressing the Mig2-GFP, Mig2*nls1*-GFP and Mig2*nls2*-GFP were grown to early-log phase (*A*_600nm_ of less than 0.8) in YEPD medium. Half of the culture was shifted to YEPGly medium for 1 h. Cells (25 μl) were loaded onto poly L-lysine-coated slides, and the remaining suspension was immediately withdrawn by aspiration. One microliter of DAPI (2.5 μg/ml in 80% glycerol) was added, and a cover slide was placed over the microscope slide. GFP and DAPI localization in cultures was monitored by direct fluorescence using a Leica DM5000B microscope. To avoid the non-linear range of fluorescent signals, cells highly overexpressing GFP-tagged fusion protein were excluded from further analyses. The localization of proteins was monitored by visual inspection of the images. At least 100 cells were scored in each of at least three independent experiments. The distribution of fluorescence was scored in the following way: N, denotes a nuclear fluorescence signal; M, mitochondrial fluorescent signal without nuclear fluorescence signal. Images representative of the results obtained are shown. Images were processed using Adobe Photoshop CS4.

### Statistical analysis

Data were obtained from at least 3 independent experiments. Results are shown as the mean ± standard error (S.E.M.).

### Preparation of crude protein extracts

Yeast protein extracts were prepared as follows: yeasts were grown in 10 to 20 ml of YEPD (H-Glc) at 28°C to an optical density at 600 nm of 0.8. Half of the culture was shifted to YEPGly (L-Glc) for 1 h. Cells were collected, washed twice with 1 ml of 1 M sorbitol and resuspended in 200 μl PBS buffer (150 mM NaCl, 100 mM Na_2_HPO_4_, 18 mM NaH_2_PO_4_, pH 7.3) containing Roche Protease Inhibitor plus 1 mM DTT and 0.1% Triton X100. The cells were broken using a FastPrep homogenizer (Thermo Electron Co.). Two pulses of 20s at 6.0 m/s were given in the presence of glass beads. Then, 200 μl of PBS buffer were added to the suspension. After centrifugation at 19,000 x g for 30 min at 4°C, the supernatant was used as crude protein extract.

### Enzyme assay

Invertase activity was assayed in whole cells as previously described [39] and expressed as micromoles of glucose released per minute and 100 mg of cells (dry weight).

### Immunoblot analysis

Mutant or wild-type yeast cells were grown to an optical density at 600 nm of 0.8 in YEPD medium containing high-glucose (2%) and shifted to low-glucose conditions for 1 h. The cells were collected by centrifugation (3,000 g, 4°C, 2 min) and crude extracts were prepared as described above. For Western blotting, 20 to 40 μg of proteins were separated by SDS-12% polyacrylamide gel electrophoresis (SDS-PAGE) and transferred to an enhanced chemiluminescence PVDF transfer membrane (Amersham Biosciences) by electroblotting. The membrane was then incubated with anti-GFP (Invitrogen), anti-Kap60 (Santa Cruz Biotech) or anti-Kap95 (Santa Cruz Biotech) as primary antibodies and the appropriate secondary antibody later. Horseradish peroxidase-conjugated protein-A was used as secondary reactant. West Pico Chemiluminescent system (Pierce) was used for detection.

### Co-immunoprecipitation assay

Immunoprecipitation experiments were performed using whole cell extracts from different strains. The extracts were incubated with anti-Kap60, anti-Kap95 or anti-Pho4 polyclonal antibodies for 3 h at 4°C. Protein A-Sepharose beads (Amersham Biosciences) were then added and incubated for 3 h at 4°C in a spinning wheel. After extensive washing with PBS plus 0.5% deoxycholate buffer, immunoprecipitated samples were boiled in SDS-loading buffer (50 mM Tris–HCl, pH 6.8, 100 mM DTT, 2% SDS, 0.1% bromophenol blue, 10% glycerol). The proteins were separated in a 12% SDS-polyacrylamide gel (SDS-PAGE), transferred to an enhanced chemiluminicence PVDF membrane and immunobloted as described above using anti-GFP monoclonal antibodies. Values shown are representative results from individual experiments.

### GST pull-down experiments

*E*. *coli* cells from the BL21(DE3) pLysS strain were transformed with the fusion protein expression vectors pGEX/MIG2 and pGEX/MIG2nls1. Cells were grown to *A*_600nm_ 0.5-0.8, induced with 0.5 mM isopropyl-1-thio-β-D-galactopyranoside at 37°C for 3 h, and collected by centrifugation. The pellet was resuspended in PBS buffer (150 mM NaCl, 100 mM Na_2_HPO_4_, 18 mM NaH_2_PO_4_, pH 7.3) and sonicated. Insoluble material was removed by centrifugation (17,000 x g for 20 min at 4°C). The soluble extract was incubated with glutathione-Sepharose 4B (Amersham Biosciences) for 1 h at 4°C, washed extensively with PBS buffer and resuspended in the same buffer. The Mig2-GST and Mig2*nls*1-GST fusion proteins coupled to glutathione-Sepharose were incubated with yeast whole cell extracts from the wild-type strain (W303-1A) for 1 h at 4°C in PBS buffer. The cell extracts were obtained from yeast cells grown in YEPD medium containing high-glucose (2%) and shifted to 0.05% glucose plus 3% glycerol (low-glucose) for 1 h. Beads were gently washed five times with 2.5 ml of PBS buffer, boiled in 25 μl sample-loading buffer, and analyzed by SDS-PAGE followed by Western blot using anti-Kap95 or anti-Kap60 antibodies and horseradish peroxidase-conjugated protein-A. Bound antibodies were detected using the West Pico Chemiluminescent system (Pierce).

GST fusion protein expression vectors pGEX-Kap60, pGEX-Kap95 and pGEX-GSP1 were transformed into *E*. *coli* strain BL21(DE3) pLysS. Cells were grown to *A*_600nm_ 0.5-0.8, induced with 0.5 mM isopropyl-1-thio-β-D-galactopyranoside at 37°C for 3 h, and collected by centrifugation. Cell pellets were resuspended in PBS buffer and sonicated. The GST-Kap60, GST-Kap95 and GST-Gsp1 fusion proteins coupled to glutathione-Sepharose beads were incubated with 2.5 units of thrombin (2 h at 4°C) for site-specific separation of the GST affinity tag from Kap60, Kap95 and Gsp1 proteins. Affinity-purified Gsp1 was immediately loaded with GTP or GDP by adding 30 mM K_2_PO_4_ (pH 7.5), 1 mM GTP or 1 mM GDP and 10 mM EDTA (pH 8.0). Then, it was incubated at room temperature for 1 h, supplemented with 20 mM magnesium acetate, incubated on ice for 30 min, and frozen at -80°C [37]. Gsp1-mediated Kap95/Kap60 interactions with Mig2 were analyzed by incubating purified Kap95 or Kap60 with GST-Mig2 bound to glutathione-Sepharose beads in the presence of Gsp1(GTP) or Gsp1(GDP) for 1 h at 4°C in PBS buffer. Beads were washed and analyzed as described above.

## Results

### The karyopherin Msn5 (Kap142) is not essential for Mig2 nuclear import

The karyopherin Msn5 is required for nuclear export of Mig1, a transcriptional repressor of *S*. *cerevisiae*[40]. Since the Msn5 protein functions both as a nuclear importin and exportin carrier [41] and Mig1 and Mig2 proteins have several common structural motifs and functions, we first tested for a possible role of Msn5 in Mig2 nuclear import.

**Figure 1:**
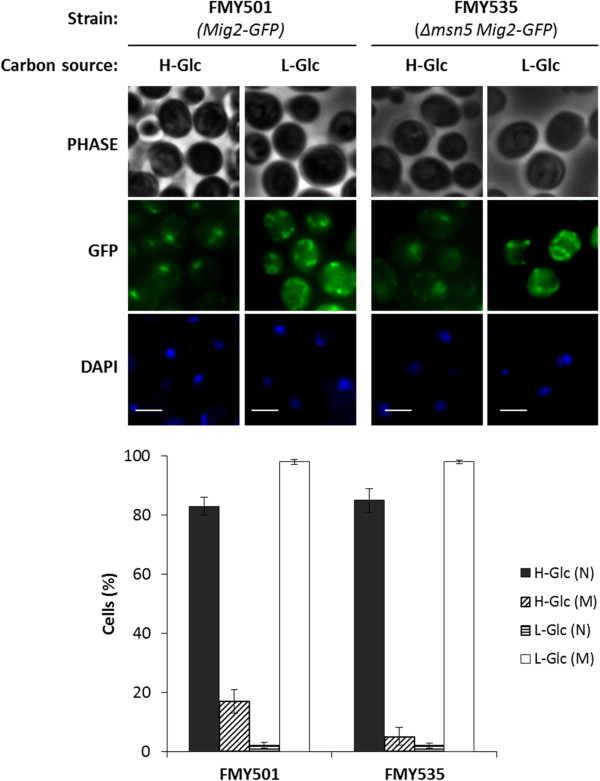
**Localization of Mig2-GFP in *Δmsn5 *yeast cells.** The FMY501(Mig2-GFP) and FMY535 (*Δmsn5* Mig2-GFP) yeast strains, were grown in YEPD high-glucose medium (H-Glc) until an *A*_600nm_ of 1.0 was reached and then transferred to YEPGly low glucose medium (L-Glc) for 5 min. Cells were stained with DAPI and imaged for GFP and DAPI fluorescence. Scale bar is 10 μm. The nuclear and mitochondrial localization of Mig2-GFP protein was determined in at least 100 cells per growth condition. No statistically significant differences were detected between the wild-type and the mutant strains. N, denotes a nuclear fluorescence signal and M, mitochondrial fluorescence signal.

Thus, nuclear accumulation of Mig2-GFP was determined in *Δmsn5* mutant cells by fluorescence microscopy, as compared to wild-type cells. The FMY501 (Mig2-GFP) and FMY535 (*Δmsn5* Mig2-GFP) strains showed identical nuclear distributions of Mig2-GFP growing in high and low glucose conditions. In high glucose conditions, nuclear accumulation of Mig2 was observed, although cytoplasmic background fluorescence was also detected and after a 5 min shift to low glucose-glycerol medium, strong green fluorescence of the characteristic branched tubular mitochondrial network was observed (Figure 1).

These results demonstrate that Mig2-GFP enters and exits from the nucleus both in the presence and absence of the Msn5 protein. This suggests that Msn5 is not implicated in either Mig2 nuclear import or export.

### Nuclear import of Mig2 is altered in a *kap95*^*ts*^ mutant strain but not in *kap60*^*ts*^ mutant cells

**Figure 2:**
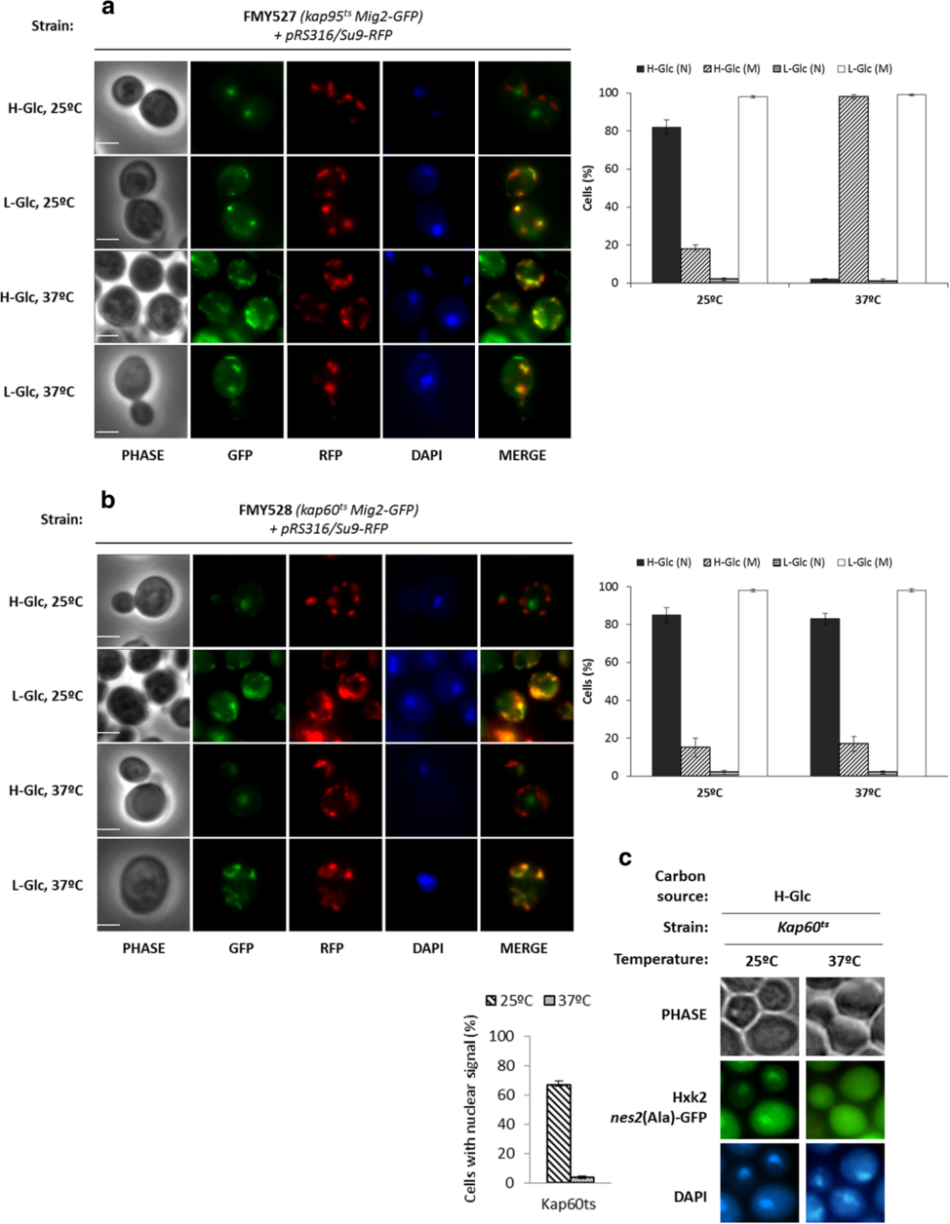
**Localization of Mig2-GFP in *kap95***^***ts***^**and *kap60***^***ts***^**yeast cells.** (**a**) The FMY527 (*kap95*^*ts*^ Mig2-GFP) and (**b**) FMY528 (*kap60*^*ts*^ Mig2-GFP) strains were transformed with plasmid pRS316/Su9-RFP. Transformed cells were grown in high-glucose synthetic medium (H-Glc) until an *A*_600nm_ of 1.0 was reached and then transferred to low glucose synthetic medium (L-Glc) for 5 min. (**c**) The JCY1410 (*kap95*^*ts*^) strain transformed with plasmid YEp352/Hxk2*nes2*(Ala)-GFP was used as positive control. Transformed cells were grown in high-glucose synthetic medium (H-Glc) until an *A*_600nm_ of 1.0 was reached. The cells were grown at 25°C (permissive temperature) and then shifted to 37°C (not permissive temperature) for 1 h. Cells were stained with DAPI and imaged for GFP, RFP and DAPI fluorescence. Scale bar is 10 μm. The nuclear or mitochondrial localization of Mig2-GFP and the nuclear or cytoplasmic localization of Hxk2*nes2*(Ala) proteins was determined in at least 100 cells per growth condition. Error bars represent standard deviations for three independent experiments. N denotes a nuclear fluorescence signal and M mitochondrial fluorescence signal.

Kap60 normally binds proteins through a highly basic stretch close to its N-terminus, resulting in the recruitment of Kap95 and formation of a heterotrimeric complex that facilitates nuclear import [36]. The carrier Kap60 recognizes two classes of NLSs matching the consensus K(K/R)X_1-3_(K/R)_1-2_ and (K/R)(K/R)X_1-3_(K/R)X_10-12_(K/R)_3-5_. The first class, known as monopartite NLS, has a single cluster of basic amino acid residues and the second class, known as bipartite NLS, has two clusters of basic amino acids separated by a 10-12 amino acid linker [42]. Since Mig2 presents two monopartite NLS motives ^32^KKRHLR^37^ and ^75^RRLKK^79^, we hypothesized that the Kap60/Kap95 complex may mediate the nuclear import of Mig2. To test this, we analyzed the subcellular localization of Mig2-GFP in temperature-sensitive *kap60*^*ts*^ and *kap95*^*ts*^ mutant cells. The *kap60*^*ts*^ and *kap95*^*ts*^ mutants were grown at the permissive temperature (25°C) in high glucose conditions and then shifted to low glucose conditions for 1 h. Then, the high and low glucose cultures were shifted to the nonpermissive temperature (37°C) for 1 h. As can be seen in Figure 2, a similar accumulation of Mig2 was detected in the nuclei and mitochondria of wild-type, *kap60*^*ts*^ and *kap95*^*ts*^ mutant cells grown at permissive temperature (Figure 1, Figure 2a and b). In high glucose conditions Mig2 mostly accumulates in the nucleus (80%) but in low-glucose conditions, Mig2 rapidly moves to the mitochondrial network (98%). RFP fused to the presequence of subunit 9 of the mitochondrial F_o_-ATPase protein was used as a mitochondrial marker and, as seen in Figure 2a, Mig2-GFP largely colocalized with Su9-RFP protein. Thus, under nutritional stress, Mig2 is essentially a mitochondria associated protein. Moreover, Mig2 translocation from the nucleus to the mitochondria is reversible, because Mig2 localizes back to the nucleus after a shift from low to high glucose conditions [25].

However, in *kap95*^*ts*^ mutant cells incubated at the non-permissive temperature (37°C) and both in high and low-glucose conditions, 98% of the Mig2 protein was located in the mitochondrial network and no nuclear accumulation of Mig2 was observed in high glucose conditions after 1 h of culture (Figure 2a). In contrast, in *kap60*^*ts*^ mutant cells incubated at the nonpermissive temperature (37°C) in high glucose conditions, 80% of cells showed nuclear accumulation of the Mig2 protein (Figure 2b), whereas if we used a positive control such as Hxk2 [36], in *kap60*^*ts*^ mutant cells incubated at the nonpermissive temperature, no nuclear accumulation of Hxk2nes2(Ala) was observed after 1 and 5 h of culture (Figure 2c). These results suggest that the Kap95 protein is necessary for nuclear import of Mig2, but the Kap60 protein does not participate in this process.

### Mapping residues necessary for NLS function

**Figure 3:**
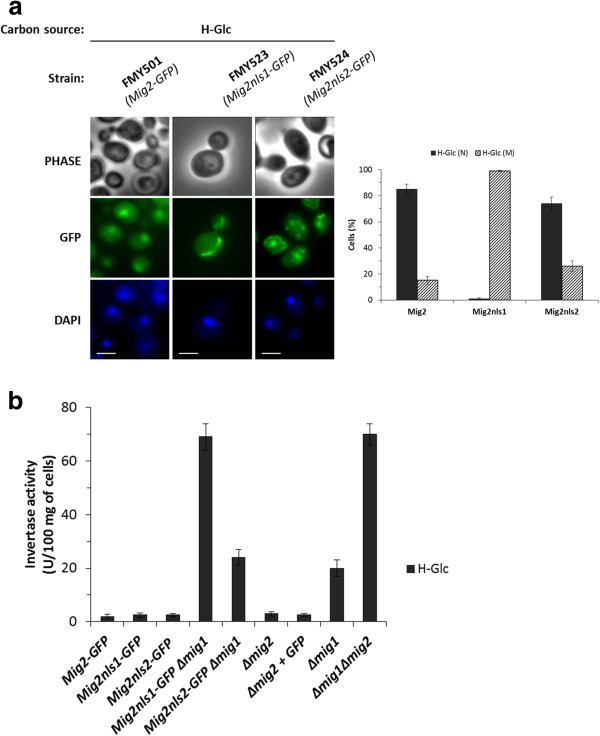
**Identification of Mig2 NLSs.** (**a**) The FMY501 (Mig2-GFP), FMY523 (Mig2*nls1*-GFP) and FMY524 (Mig2*nls2*-GFP) were grown in YEPD high-glucose medium (H-Glc) until an *A*_600nm_ of 1.0 was reached and then transferred to YEPGly low glucose medium (L-Glc) for 5 min. The cells were visualized by fluorescence microscopy, DAPI staining revealed nuclear DNA. Scale bar is 10 μm. The nuclear localization of fluorescent reporter proteins was determined in at least 100 cells in three independent experiments. Means and standard deviations are shown for at least three independent experiments. N denotes a nuclear fluorescence signal and M mitochondrial fluorescence signal. (**b**) The FMY501 (Mig2-GFP), FMY523 (Mig2*nls1*-GFP), FMY524 (Mig2*nls2*-GFP), FMY525 (Mig2*nls1*-GFP *Δmig1*), FMY526 (Mig2*nls2*-GFP *Δmig1*), Y14575 (*Δmig2*), FMY536 (*Δmig2* + GFP), H174 (*Δmig1*) and MAP24 (*Δmig1 Δmig2*) strains were grown in YEPD high-glucose medium (H-Glc) until an *A*_600nm_ of 0.8 was reached. Invertase activity was assayed in whole cells. Values are the averages of results obtained in four independent experiments.

We next determined whether the two putative Mig2 NLS sequences are necessary for the entry of Mig2 into the nucleus. In order to investigate this, the putative NLS sequences were mutated and tested for their function. The presence of basic amino acids such as lysine and arginine appears to be an important feature of the NLSs [42]. Thus, we created two *MIG2GFP* mutants in which three basic residues of either NLS1 or NLS2 were replaced by alanine residues to generate *MIG2K32AK33AR37AGFP* (encoding Mig2*nls1*-GFP) and *MIG2R75AR76AK79AGFP* (encoding Mig2*nls2*-GFP). Analysis of the intracellular localization of the different Mig2-GFP variants by fluorescence microscopy showed that the Mig2*nls2*-GFP fusion protein accumulated in the nuclei (74%) in high-glucose conditions (Figure 3a). However, in high glucose conditions the Mig2*nls1*-GFP protein was excluded from the nuclei and a mitochondrial (99%) accumulation was detected (Figure 3a). Thus, the *nls1* mutation causes a strong defect in the nuclear import of Mig2.

Accordingly, we hypothesize that consistent with the inability of the mutant Mig2*nls1* protein to enter into the nucleus *in vivo* (Figure 3a); the mutants lacking the NLS1 must be unable to signal glucose repression in a *Δmig1* mutant strain. Thus, we investigated the Mig2-dependent glucose signaling function in the presence and absence of the Mig1 protein (Figure 3b). The cells were grown in YEPD complex medium to an *A*_600nm_ of 0.8 and the exocellular invertase activity was determined in whole cells as a glucose repression marker. The specific activity of invertase shows identical strong derepression in both *Δmig1Δmig2* and *Δmig1Mig2nls1* mutant strains. However, in the Δ*mig1Mig2nls2* mutant strain a derepression level similar to the *Δmig1* strain was observed. These results confirm the above finding, that the strain expressing Mig2*nls1* has lost nuclear import capacity and thus cannot exert its function as a transcriptional repressor.

### Mig2 interacts with Kap60 and Kap95

**Figure 4:**
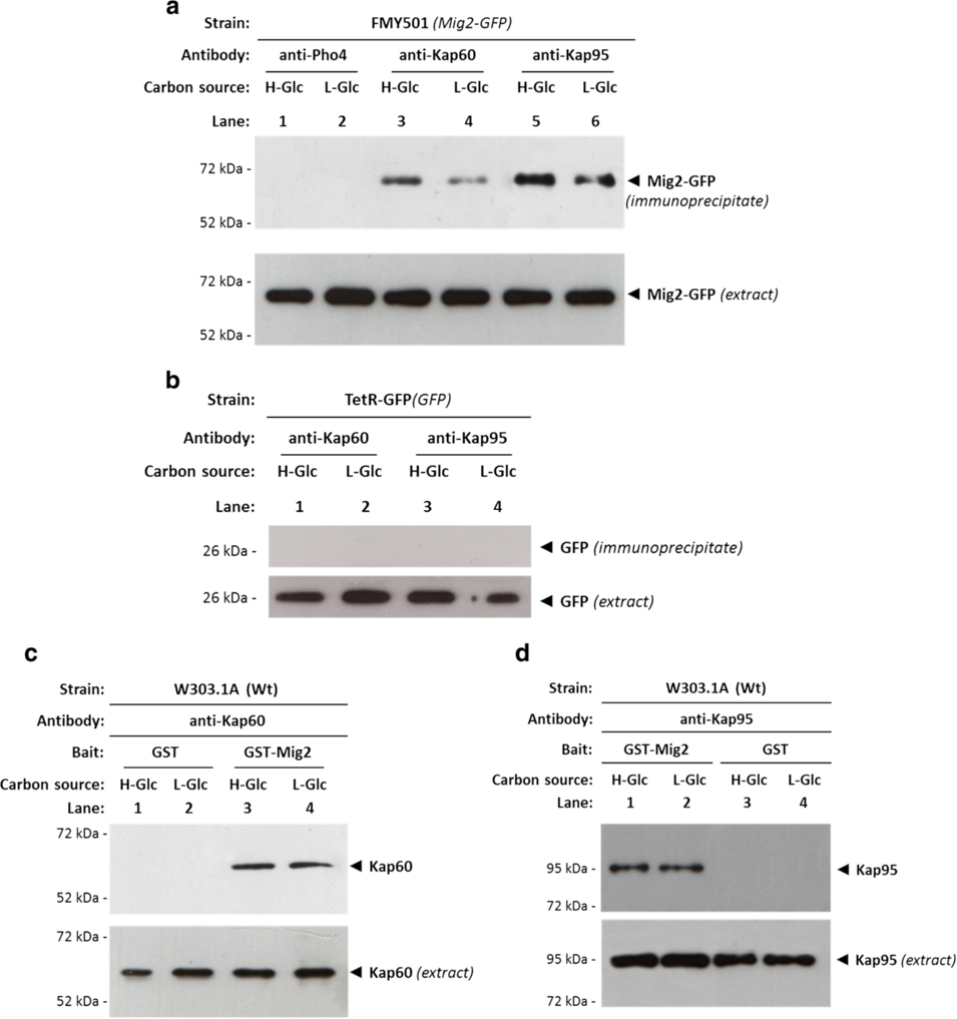
**Interaction of Kap60 and Kap95 with Mig2. ***In vivo * co-immunoprecipitation of Kap60 and Kap95 with Mig2. The wild-type, FMY501 (Mig2-GFP) (**a**) or a control strain, (TetR-GFP) synthetizing GFP only (**b**) were grown in YEPD-media until an *A*_600nm_ of 0.8 was reached and then shifted to low glucose (L-Glc) conditions for 1 h. The cell extracts were immunoprecipitated with a polyclonal anti-Kap60 or anti-Kap95 antibody (lanes 3-6) or a polyclonal antibody against Pho4 (lanes 1 and 2). Immunoprecipitates were separated by 12% SDS-PAGE, and co-precipitated Mig2-GFP was visualized by Western blot with polyclonal anti-GFP antibodies. The level of immunoprecipitated Kap60 or Kap95 in the blotted samples was determined by using anti-Kap60 and anti-Kap95 antibodies, respectively. The level of Mig2-GFP and GFP present in the different extracts was determined by Western blot using anti-GFP antibody. The Western blots shown are representative of results obtained from four independent experiments. The GST-Mig2 fusion protein was purified on glutathione-Sepharose columns. Equal amounts of GST-Mig2 were incubated with cell extracts from the wild-type strain W303-1A. The yeasts were grown in YEPD media until an *A*_600nm_ of 0.8 was reached and then shifted to low (L-Glc) glucose conditions for 1 h. After exhaustive washing the proteins were separated by 12% SDS-PAGE, and retained Kap60 and Kap95 proteins were visualized by Western blot using polyclonal anti-Kap60 (**c**) and anti-Kap95 (**d**) antibodies respectively. For the control samples, GST protein was also incubated with high- (H-Glc) and low-glucose (L-Glc) cell extracts, but no signals were detected. The level of Kap60 and Kap95 proteins present in the different extracts used in Figure 4c and 4d was determined by Western blot using anti-Kap60 and anti-Kap95 antibodies respectively. The Western blots shown are representative of results obtained from four independent experiments.

To test whether Mig2 binds to Kap60 and Kap95 *in vivo* we carried out an immunoprecipitation assay on cells expressing Mig2-GFP and GFP as control. Cell extracts from FMY501 (Mig2-GFP) or TetR-GFP were immunoprecipitated with anti-Kap60 and anti-Kap95 antibodies. The resulting immunoprecipitates were assayed for the presence of Mig2-GFP or GFP by immunoblot analysis with anti-GFP antibodies. As shown in Figure 4a, specific signals of Mig2-GFP were observed with samples immunoprecipitated with both anti-Kap60 and anti-Kap95 antibodies in the strain expressing Mig2-GFP either in high and low glucose conditions. However, no signals were observed when the experiment was done using the strain expressing GFP although similar amounts of GFP were detected in the immunoprecipitates when anti-GFP antibody was used (Figure 4b). These results indicated that the glucose levels do not affect the affinity of Mig2, since the Mig2-GFP interaction with Kap60 and Kap95 proteins was similar with samples from high and low glucose-grown cultures. Thus, our data suggest that the glucose levels do not affect the affinity of Mig2 for the importin-α and β.

To confirm the *in vitro* interaction of Mig2 with Kap60 and Kap95, we also performed a GST pull-down assay. We used crude protein extracts from a wild-type strain and a GST-Mig2 fusion protein expressed in *E*. *coli*. As shown in Figure 4, a clear retention of Kap60 protein (Figure 4c) and Kap95 (Figure 4d) was observed both in high and low-glucose conditions. When a control with GST protein in the reaction mix was used, no Kap60 or Kap95 signal was observed. These results also confirm that the interaction between Mig2 and both importins is similar under high and low-glucose conditions and suggest that both proteins interact in a glucose independent manner.

**Figure 5:**
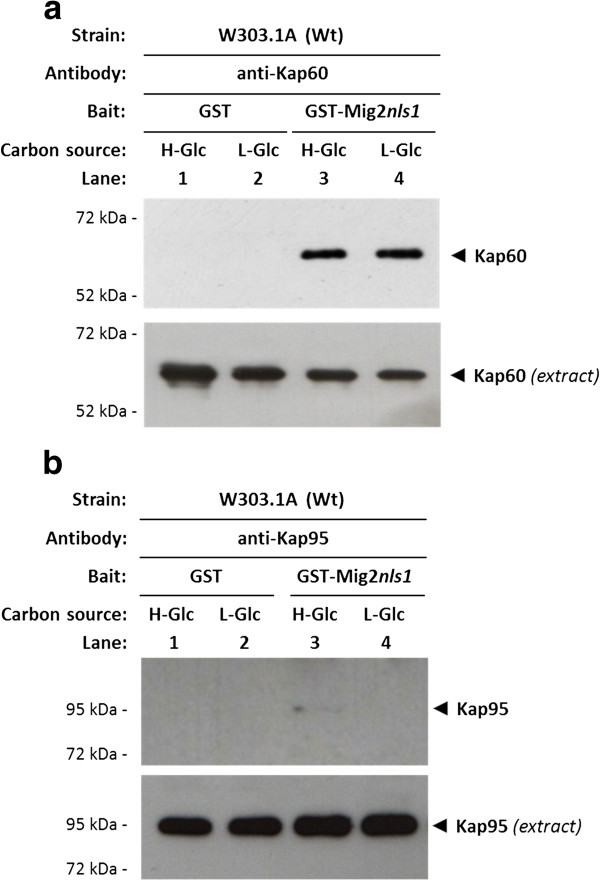
**Interaction of Kap60 and Kap95 with Mig2 *nls1. *** The GST-Mig2*nls1*fusion protein was purified on glutathione-Sepharose columns. Equal amounts of GST-Mig2*nls1* were incubated with cell extracts from the wild-type strain W303-1A. The yeasts were grown in YEPD media until an *A*_600nm_ of 0.8 was reached and then shifted to low (L-Glc) glucose conditions for 1 h. After exhaustive washing the proteins were separated by 12% SDS-PAGE, and retained Kap60 and Kap95 proteins were visualized on a Western blot with polyclonal anti-Kap60 (**b**) and anti-Kap95 (**c**) antibodies respectively. For the control samples, GST protein was also incubated with high- (H-Glc) and low-glucose (L-Glc) cell extracts, but no signals were detected. The level of Kap60 and Kap95 proteins present in the different extracts used in Figure 5a and 5b was determined by Western blot using anti-Kap60 and anti-Kap95 antibodies respectively. The Western blots shown are representative of results obtained from four independent experiments.

To analyse the interaction of Mig2*nls1* mutant protein with Kap60 and Kap95, we also performed a GST pull-down assay. We used crude protein extracts from a wild-type strain and purified GST-Mig2*nls1* fusion protein expressed in *E*. *coli*. As shown in Figure 5a, a clear retention of Kap60 protein was observed both in high and low-glucose conditions in the sample containing GST-Mig2*nls1* and crude extract from the wild-type strain. However, a very weak or no retention of Kap95 protein was observed for the sample containing GST-Mig2*nls1* and crude extract from the wild-type strain (Figure 5b). Together, these data suggest that the Mig2-Kap95 interaction strongly depends on the NLS1 motif, whereas the latter has no influence on the interaction with Kap60.

### The interaction between Kap95 and Mig2 requires auxiliary factors other than Kap60 protein

**Figure 6:**
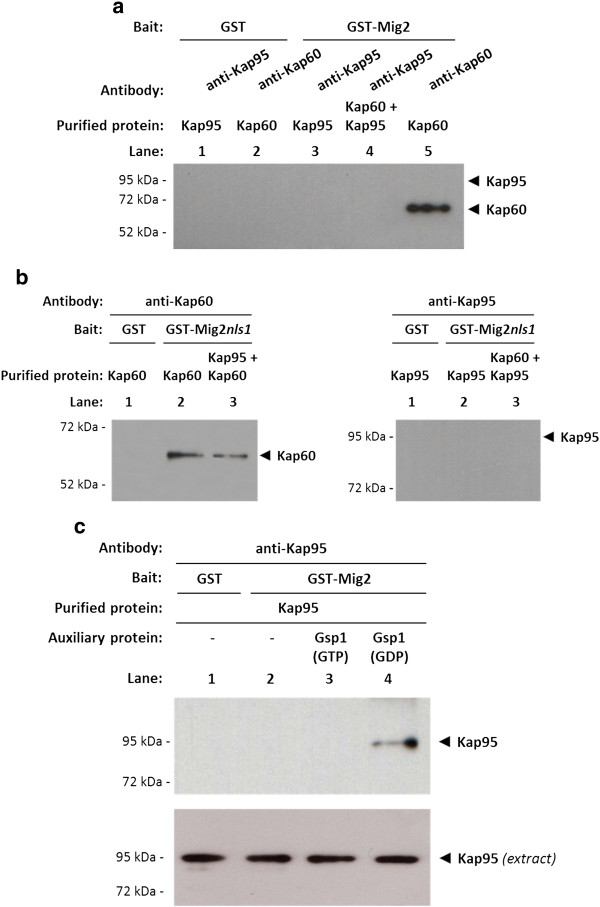
**The import Mig2-Kap95 complex formation *in vitro. *** GST pull-down analysis of Mig2 (**a**) and Mig2*nls1* (**b**) interaction with Kap60 and Kap95 purified proteins. (**c**) Effect of Gsp1 on the Mig2-Kap95 interaction stability. GST-Mig2, GST-Mig2*nls1*, GST-Gsp1, GST-Kap60 and GST-Kap95 fusion proteins were purified on glutathione-Sepharose columns and incubated with thrombin to release, respectively, Gsp1, Kap60 and Kap95 proteins. The Gsp1 protein was loaded with GTP to generate Gsp1(GTP) or with GDP to generate Gsp1(GDP). The purified proteins were incubated with purified GST-Mig2 or GST-Mig2*nls1* bound to glutathione-Sepharose beads in the absence (**a** and **b**) or in the presence (**c**) of Gsp1(GTP) and Gsp1(GDP). The beads were washed extensively. Co-precipitated proteins were resolved by 12% SDS-PAGE and visualized on a Western blot with polyclonal anti-Kap60 or anti-Kap95 antibodies. The level of Kap95 protein present in the different extracts used in Figure 5c was determined by Western blot using anti-Kap95 antibodies. The Western blots shown are representative of results obtained from four independent experiments.

As indicated above, Kap95 is required for efficient nuclear import of Mig2. In order to address the question of if this function is direct or indirect, we determined if Kap60 is needed to mediate the interaction with Mig2. For this purpose, again purified proteins from E. coli were employed. We used GST-Mig2, GST-Mig2nls1, GST-Kap60 and GST-Kap95 fusion proteins. The GST fusion proteins were immobilized with glutathione-Sepharose and released with thrombin. First, we immobilized GST-Mig2 on glutathione-Sepharose beads and analysed the binding of purified Kap60 and Kap95 proteins. Our results confirmed that purified Kap60 has a strong affinity for Mig2 (Figure 6a, lane 4) and that this interaction was not NLS1-dependent (Figure 6b). However, no interaction of Mig2 or Mig2*nls1* with Kap95 was detected using purified proteins either in the presence or absence of Kap60 (Figure 6b).

These results indicate that auxiliary factors other than Kap60 protein are required for the Mig2-Kap95 interaction. In order to investigate if the Gsp1-GTP/GDP cycle controls the formation and/or the transport direction of the Mig2-Kap95 complex [43], we tried to reconstruct the import complex *in vitro* using purified proteins. To study how the different factors affect the Mig2-Kap95 interaction affinity, we performed GST pull-down experiments with purified proteins. We used GST-Mig2, GST-Gsp1 and GST-Kap95 fusion proteins from *E*. *coli* lysates. The GST fusion proteins were immobilized with glutathione-Sepharose and released with thrombin. First, we immobilized GST-Mig2 on glutathione-Sepharose beads and analysed the binding of Kap95. Our results indicate that purified Kap95 has no affinity for Mig2 (Figure 6c, lane 2). However, as expected for an importin-type receptor, the complex formation between Kap95 and Mig2 is regulated by the guanine nucleotide-binding protein Gsp1. Our data indicate that the Mig2-Kap95 complex is not formed in the presence or absence of Gsp1-GTP (Figure 6c lanes 2 and 3). However, the presence of Gsp1-GDP facilitates the formation and increases the stability of the Mig2-Kap95 complex (Figure 6c lane 4).

This experiment suggests that a ternary complex between Kap95, Gsp1 and Mig2 is formed *in vitro*. Moreover, the complex formation is controlled by the GDP/GTP-bound state of Gsp1, because loading of Gsp1 with GTP disrupts the formation of the import complex and Gsp1 loaded with GDP acts as an auxiliary factor required for Mig2-Kap95 interaction and for complex stabilization.

## Discussion

Glucose levels regulate Mig2 translocation from the nucleus to the mitochondria [25]. In high-glucose conditions, nuclear Mig2 interacts with Mig1 and Hxk2 to participate in the *SUC2*-Mig1 repressor complex. In low glucose conditions, Mig1 S311 is phosphorylated by Snf1 kinase [21]. Once phosphorylated Mig1 leaves the nucleus [44]. Nuclear Hxk2 loses its anchoring protein and also moves into the cytosol [24]. This process leads to *SUC2* repressor complex dissociation and *SUC2* gene derepression. In these conditions, the vast majority of Mig2 accumulates in the mitochondria. The mitochondrial targeting of Mig2 in low glucose conditions is Ups1-dependent [25]. While it is essential for Mig2 to enter the nucleus to perform its role as transcriptional repressor, the mechanism by which the protein transverses the nuclear envelope has not been investigated until now. In the current study, we have extended our knowledge of the nucleocytoplasmic shuttling of Mig2 by showing that its nuclear translocation is mediated by the importin-β (Kap95) and regulated by the Ran GTPase protein (Gsp1).

In this study, we provide evidence that nuclear import of Mig2 is independent of the yeast Msn5 karyopherin and that Kap95 is the carrier responsible for the nuclear accumulation of Mig2 under high-glucose conditions. We observe that Mig2 protein in *Kap95*^*ts*^ mutant cells is not efficiently imported into the nucleus and accumulates in the mitochondrial tubules during high-glucose growth. However, in *Kap60*^*ts*^ mutant cells Mig2 is efficiently imported into the nucleus in high-glucose conditions. These data suggest that Kap95 is the karyopherin responsible for nuclear import of the Mig2 protein, either independently or as part of a larger complex from which Kap60 is excluded.

Taken together, our results demonstrate that the Lys32, Lys33 and/or Arg37 are critical amino acid residues in the Mig2 NLS1 sequence. Moreover, since the NLS2 mutation does not affect Mig2 import because it does not decrease significantly its nuclear accumulation and glucose repression signalling, our data suggest that only the NLS1 motif is necessary for efficient nuclear import of Mig2-GFP *in vivo*. These results are surprising because it has not been previously described that Kap95 directly recognizes a conventional NLS. However, the results are consistent with the idea that only Kap95 protein participates in Mig2 nuclear import. Moreover, the Mig2*nls1* mutant protein lacks repressor function, while, the Mig2*nls2* mutant protein complements the *Δmig2* mutation in a double *Δmig1Δmig2* mutant and exhibits predominantly nuclear localization. Our pull-down assays employing purified proteins suggest, that the Mig2-Kap95 complex is stable and does not require Kap60 for association in the presence of Gsp1(GDP), as happens in the cytoplasm. However, in the nucleus the complex is disrupted in the presence of high levels of Gsp1(GTP). Thus, efficient dissociation of the Mig2-Kap95 complex is mediated primarily by Gsp1(GTP) which displaces Mig2 from the importin-β receptor. These experiments, also exclude the possibility that some unknown protein/s in the yeast extract could mediate the interaction between the Mig2 protein and the Kap95 protein.

Importin-β (Kap95) has two distinct mechanisms by which it associates with transport substrates: indirect and direct. The more conventional import pathway takes place by the indirect association of cargo with the Kap60-Kap95 (importin-α/β) complex. While Kap60 binds to a classical NLS present in the cargo, Kap95 associates with Kap60 and is primarily responsible for translocation of the complex across the nuclear pore. Conversely, direct association involves Kap95 directly binding to its cargo. Several examples of this direct mechanism have been described in mammalian cells [15,45-47], implying that the importin-β (Kap95) may be able to function independently of Kap60 as a nuclear import receptor. The findings of the present study not only establish that Mig2 is similar to the above mammalian proteins in being recognized by Kap95 alone but also demonstrate conclusively for the first time in yeast that Kap95, in concert with Gsp1, is sufficient to mediate nuclear import of Mig2 in the absence of Kap60. The Mig2 protein has been shown to bind directly to Kap95 in the presence of Gsp1(GDP) without requiring Kap60 for association. Thus, our results are in accordance with previous publications describing Kap95 as an essential karyopherin in the nuclear import of several proteins without requiring importin-α [13,14].

Although the details are not fully understood, there are novel NLS-dependent nuclear import pathways exclusively mediated by Kap95 that are presumably analogous to those mediated by other importin-β homologs. The Kap95 protein has been described as contributing to the nuclear import of cargos by recognizing import signals different from the classical NLS [16,48,49]. Surprisingly, a classical NLS-mediated transport mechanism is required for Mig2-Kap95 binding and Mig2 nuclear accumulation in the absence of a functional Kap60 protein.

Our results also indicate that Kap60 interacts with Mig2-GFP both in high and low-glucose conditions as demonstrated by immunoprecipitation and GST-pull down experiments. However, this interaction is not required for Mig2-GFP nuclear import. Moreover, the NLS1 sequence of Mig2 is not involved in the Kap60-Mig2 interaction. Thus, the physiological significance of Mig2-Kap60 is unknown at the current stage. While a number of nuclear import pathways have been identified, the definition of general rules of what constitutes the targeting signal and the role of Kap60 has only been possible for the classical nuclear import pathway. For other pathways, such rules have not yet been identified probably because an insufficient number of cargos are known, the recognition of cargo by the carrier requires further characterization by structural and interaction analyses or no common rules exist and the recognition is specific to specific cargos. The key to progress is to integrate seemingly disparate types of information, using structural, and sequence information and protein-protein interactions.

## Conclusions

The current study has identified a new pathway for Mig2 import into the nucleus. Here we provide data that support a model for Mig2 nuclear import that includes Kap95 as the predominant karyopherin and the NLS1 of Mig2 as essential for efficient Mig2-Kap95 interaction and nuclear import. Mig2, in the presence of Gsp1(GDP), directly binds importin-β (Kap95), and together they form a complex to allow the passage of Mig2 through the nuclear pore. Thus, we found that Kap95 forms a stable complex with Mig2 in the cytoplasm where the level of Gsp1(GDP) is high. After translocation into the nucleus, where the Gsp1(GDP) level is low and the Gsp1(GTP) level is high, this complex is dissociated via a mechanism that includes GDP/GTP exchange in the import complex.

## Abbreviations

NLS: Nuclear localization sequence; GST: Glutathione S-transferase; GFP: Green fluorescent protein; RFP: Red fluorescent protein; DAPI: 4’,6-Diamino-2-phenylindole.

## Competing interests

The authors declared that they have no competing interests.

## Authors’ contributions

AF-C carried out the experiments, analysed and interpreted the data. MV purified proteins from bacteria and did *in vitro* experiments. PH made important contributions making several DNA constructs and the design of experiments. FM was involved in the planning, experimental design, data analysis and wrote the manuscript. All authors read and approved the final version of the manuscript.
